# Transgender women in Kenya experience greater stigma, depressive symptoms, alcohol and drug use and risky sexual practices than cis-gendered men who have sex with men

**DOI:** 10.1186/s12889-023-16348-6

**Published:** 2023-08-05

**Authors:** Supriya D. Mehta, Fredrick O. Otieno, Joshua Kimani, Elizabeth Wahome, Duncan Okal, Abhishikta Roy, Elise van der Elst, Susan M. Graham, Eduard J. Sanders, Robert C. Bailey

**Affiliations:** 1 Division of Epidemiology & Biostatistics, University of Illinois Chicago School of Public Health, 1750 W. Harrison Street, Jelke 1121, Chicago, IL 60612 USA; 2 Division of Infectious Disease Medicine, Rush University College of Medicine, Chicago, USA; 3 Nyanza Reproductive Health Society, Kisumu, Kenya; 4 Partners for Health and Development in Africa, Nairobi, Kenya; 5 University of Manitoba, Winnipeg, Canada; 6 Kenya Medical Research Institute-Wellcome Trust Research Programme, Kilifi, Kenya; 7 Global Health Department, University of Amsterdam, Amsterdam, The Netherlands; 8 Departments of Global Health, Medicine, and Epidemiology, University of Washington, School of Medicine, Seattle, WA USA; 9 University of Oxford, Oxford, UK; 10 Aurum Institute, Johannesburg, South Africa

**Keywords:** Transgender women, Men who have sex with men, Stigma, Depressive symptoms, Substance use, Alcohol use, Kenya

## Abstract

**Background:**

Worldwide, sexual and gender minority individuals have disproportionate burden of HIV. There are limited quantitative data from sub-Saharan Africa on the intersection of risks experienced by transgender women (TGW) in comparison to cis-men who have sex with men (MSM). This analysis addresses this gap by comparing reported stigma, psychosocial measures of health, and sexual risk practices between TGW and cis-MSM in Kenya.

**Methods:**

We analyzed data from the baseline visit of an ongoing prospective cohort study taking place in three diverse metropolitan areas. Eligible participants were HIV-negative, assigned male at birth, ages 18–29 years, and reported anal intercourse in the past 3 months with a man or TGW. Data collected by audio computer assisted self-interview included sociodemographic measures, and sexual practices occurring in the past 3 months. Multivariable regressions assessed differences between TGW and cis-MSM in selected sexual practices, depressive symptoms, alcohol and drug use, and stigma.

**Results:**

From September, 2019, through May, 2021, 838 participants were enrolled: 108 (12.9%) TGW and 730 (87.1%) cis-MSM. Adjusting for sociodemographic variables, TGW were more likely than cis-MSM to report: receptive anal intercourse (RAI; adjusted prevalence ratio [aPR] = 1.59, 95% CI: 1.32 – 1.92), engaging in group sex (aPR = 1.15, 95% CI: 1.04 – 1.27), 4 or more male sex partners (aPR = 3.31, 95% CI: 2.52 – 4.35), and 3 or more paying male sex partners (aPR = 1.58, 95% CI: 1.04 – 2.39). TGW were also more likely to report moderate to severe depressive symptoms (aPR = 1.42, 95% CI: 1.01 – 1.55), and had similar alcohol and drug abuse scores as cis-MSM. In sensitivity analysis, similar to TGW, male-identifying individuals taking feminizing gender affirming therapy had an increased likelihood of reporting RAI and group sex, and greater numbers of male sex partners and paying male sex partners relative to cis-MSM.

**Conclusions:**

Across three metropolitan areas in Kenya, TGW were more likely to report depressive symptoms and increased sexual risk taking. We identified a need for research that better characterizes the range of gender identities. Our analysis affirms the need for programmatic gender-affirming interventions specific to transgender populations in Kenya and elsewhere in Africa.

**Supplementary Information:**

The online version contains supplementary material available at 10.1186/s12889-023-16348-6.

## Introduction

Worldwide, sexual and gender minority individuals have disproportionate burden of HIV and sexually transmitted infections (STI). Gay, bisexual, and other cis-gender men who have sex with men (cis-MSM) in sub-Saharan Africa have 2-15 times higher HIV prevalence than the general male population [1]. The prevalence of HIV among cis-MSM in Kenya is estimated at 18% [2]. In our studies, 65% of cis-MSM also have sex with women [3, 4], at least in part to remain hidden in a country where homosexuality is criminalized, and in response to pervasive stigma and discrimination [5, 6]. These disparities are further exacerbated for transgender women (TGW), although data are scarce. Among a sample of 14 Kenyan TGW, HIV incidence over one year was 20.6 per 100 person-years (PY), compared to 4.5 per 100 PYs among 42 cis-MSM who reported only male partners, and 3.4 per 100 PYs among 112 cis-MSM who have sex with men and women [7]. This very high HIV incidence in TGW is in keeping with meta-analysis showing that TGW face up to 48 times the odds of HIV prevalence than the general adult population, in both high- and low- income countries [8].

Stigma, discrimination, impeded economic and educational opportunity, legal and social rights constraints, and limited access to HIV prevention and treatment synergistically intersect to increase TGW’s vulnerability to HIV even more so than for cis-MSM [9]. This intense marginalization and discrimination, with concomitant lack of healthcare and other services, is often syndemic with elevated depressive symptoms, hazardous alcohol and drug use, and elevated sexual risks [10, 11]. Data that would inform programming and services to meet the specific needs of TGW are often limited due to combining data from TGW with cis-MSM or excluding TGW from sexual and reproductive health studies altogether [12]. Within Kenya and other sub-Saharan African countries, there is limited quantitative data on the intersection of risks experienced by TGW in comparison to cis-MSM. In the TRUST/RV368 study, among 2,795 participants in Nigeria, 80.8% were cis-MSM, 10.2% were TGW, and 9.0% non-binary/other [13]. TGW had higher prevalence of HIV and gonorrhea than cis-MSM, and were more likely than cis-MSM to report being affected by stigma and engaging in receptive anal intercourse. However, information was lacking on how sexual practices correlated with psychosocial syndemics, which would allow for better understanding of the factors that contribute to increased HIV risk among TGW, and to develop responsive services. Another challenge in generating knowledge about the risks and challenges faced by cis-MSM and TGW stems from lack of clarity in how sex, gender, and gender identity are categorized. Scholars have raised conceptual issues regarding the application of “foreign origin” or "Eurocentric” terminology to the African setting, where sexual and gender identity may have unique cultural origins that contribute to temporal fluidity, varying expression, and how and whether one labels oneself a particular way [14–16].

The purpose of this analysis was to address these gaps by comparing baseline reported stigma, psychosocial measures of health, and sexual risk practices between TGW and cis-MSM participating in a prospective cohort study in Kenya. We focused on examining potential differences in known syndemics of depressive symptoms, stigma, hazardous alcohol and drug use, and sexual practices given their frequent co-occurrence and the implications for comprehensive care [11]. We hypothesized that similar to studies of cis-MSM and TGW in other settings [16], (1) stigma, depressive symptoms, and hazardous alcohol and drug use would be positively correlated, and (2) TGW would experience heightened occurrence of stigma, depressive symptoms, and hazardous alcohol and drug use as compared to cis-MSM.

## Methods

This study was approved by the ethical review boards of Kenya Medical Research Institute (SERU 3788) and Rush University (22080202). All participants provided written informed consent in their language of choice (English, Kiswahili, DhoLuo). The study was conducted in accordance with the Declaration of Helsinki.

### Recruitment and eligibility

Participants for this analysis were enrolled in *Tatu Pamoja* (“Three Together” in Kiswahili), a collaborative study led by members of the Kenya MSM Health Research Consortium (https://msmhealthresearch.org). Participants were recruited in three sites: Kisumu (western Kenya), Nairobi (central Kenya; country capital); and coastal Kenya (towns of Malindi and Mtwapa). To be eligible, participants had to be HIV-negative, report being assigned male at birth, aged 18-29 years, and report anal intercourse in the past 3 months with a cis-male or TGW. Participants were recruited through existing HIV prevention programs for cis-MSM and TGW at sites that had previously collaborated on research into mental health and substance use [17]. A total of 900 participants were to be recruited, with 300 from Kisumu and Nairobi sites and 300 from the coastal sites. Participants were enrolled in Kisumu, Nairobi, and Mtwapa between September 19, 2019, and February 20, 2020. For Malindi, there were two recruitment periods: September 19 through December 4, 2019, and December 8, 2020, through May 4, 2021. Participants were followed quarterly for up to 3 years, through December 2022.

### Data collection

Data were collected by audio computer assisted self-interview (ACASI) in the participants’ preferred language (English, DhoLuo, or Kiswahili). Data collection included demographics, socioeconomic measures, and assessment of sexual practices. Most sexual practices were assessed with a recall period of “past 3 months,” following the quarterly study visits, with some questions assessing practices at the last sexual encounter. Several psychometric scales were employed. The Patient Health Questionnaire 9 (PHQ-9) was used to assess depressive symptoms, using a cutoff score of ≥10 for moderate, moderately severe, and severe symptoms [18]. Potentially harmful or hazardous alcohol use was measured using the Alcohol Use Disorders Identification Test (AUDIT), using the cutoff score of ≥8 for potentially harmful alcohol use [19]. Drug abuse was measured using the Drug Abuse Screening Test (DAST), with a cutoff of ≥3 reflecting moderate, substantial, or severe substance abuse [20–22]. The Childhood Experience of Care and Abuse (CECA) questionnaire assessed physical abuse and unwanted sexual experiences in childhood [23]. These scales have performed well in terms of internal validity and reliability in our previous studies with Kenyan cis-MSM [17, 20, 24]. Sexual stigma was measured using a modified version of the MSM Stigma Scale [24, 25] and captured perceived and experienced stigma, with greater scores reflecting greater stigma. Interpersonal violence was assessed with three questions assessing emotional, physical, and sexually abusive behavior within a relationship, and two questions regarding physical assault/attack or rape, not specifically within the context of a relationship [26]. Details of these scores and their performance are available in our prior publications [4, 20, 24]; in Supplemental Table 3, we report Cronbach’s alpha and question alterations.

### Statistical analysis

This analysis compared selected characteristics between TGW and cis-MSM participants at their baseline visit. Gender was characterized based on two questions: (1) “What sex were you assigned at birth?” followed by (2) “What is your current gender”? Those reporting “female” or “transgender female” to the second question were classified as TGW. Self-reported gender assigned at birth and current gender were collected independently at eligibility screening and for participants who enrolled, thus allowing for discrepancy in responses. Among 851 participants enrolled in *Tatu Pamoja*, 850 provided information on gender assigned at birth: 843 (99.1%) reported being assigned male at birth and 7 (0.8%) reported being assigned female at birth, with one participant refusing. As current gender, 733 (86.1%) respondents reported being male, and 105 (12.3%) reported being female (*n*=23) or transgender female (*n*=82), while 12 (1.4%) reported “Don’t Know” or “Other,” and 1 refused. After enrollment, all participants underwent medical history and physical examination to assess sexually transmitted infections. No participants had a natal vagina or neovagina. Three of the 733 participants who reported being currently male gender, reported being assigned female at birth and were categorized as TGW for this analysis. Of note, two of these three individuals reported taking feminizing gender affirming therapy (GAT); one individual who responded “no” to taking GAT in a later question reported taking GAT obtained from a public hospital. Combining the two variables, there were 730 (87.1%) participants who were categorized as cis-males and 108 (12.9%) who were categorized as TGW. The 13 (1.5%) participants who reported “Other” gender, “Don’t Know”, or “Refused” were excluded from these analyses. As a sensitivity analysis to maximize identification of TGW, the 59 participants who reported being assigned male at birth and having current gender “Male”, but also reported taking feminizing hormones, anti-androgens, or using silicone implants, were grouped with TGW in comparison to cis-MSM (Supplemental Tables 1 and 2). Of note, the survey question that assessed use of these therapies was worded as “Which of the following gender-affirming therapies have you used or are you currently using?” We therefore maintain this question structure in this manuscript (i.e., “gender-affirming” therapy), and cannot distinguish between past use and current use.

In this cross-sectional analysis, we conducted three analyses: (1) Comparing selected sexual practices, alcohol use, drug use, depressive symptoms, and sexual stigma between TGW and cis-MSM; (2) Comparing correlations of psychosocial factors and sexual practices between TGW and cis-MSM; (3) Comparing the burden of syndemic psychosocial outcomes (elevated depressive symptoms, elevated stigma, hazardous alcohol and drug use) between TGW and cis-MSM.

In the first analysis, we compare frequency distributions between TGW and cis-MSM with chi-square tests of significance for differences in categorical variables (Fisher’s exact test applied when cell sizes were < 5) and Wilcoxon rank sum test for non-normally distributed continuous variables. To understand whether selected sexual practices (receptive anal intercourse (RAI), number of male sex partners, number of paying male sex partners, having any paying female sex partner, group sex), harmful or hazardous drinking, harmful or hazardous drug use, moderate or greater depressive symptoms, and sexual stigma differed between TGW and cis-MSM, we conducted a series of regressions with cluster-based variance estimation by study site, to account for within site clustering of participant characteristics. We conducted crude analyses and then multivariable analyses adjusted for age, educational attainment, employment status, and study site. We used log binomial regression for all binary outcomes except for RAI because it did not converge; negative binomial regression was used as the alternative. For multinomial outcomes (number of sex partners), we used multinomial logistic regression with prevalence ratio transformation. For continuous outcomes (sexual stigma score, PHQ-9 score, AUDIT score, DAST score) we used linear regression.

In the second analysis, to explore the relationship between psychosocial variables and sexual behavior, we estimated the correlation of psychometric variables (continuous scores for depressive symptoms, alcohol and drug use, sexual stigma) with the selected sexual behaviors (RAI, number of male sex partners, number of paying male sex partners, group sex, any paying female sex partner) separately for TGW and cis-MSM. Lastly, we used multinomial logistic regression to examine TGW relative to cis-MSM in relation to having syndemic outcomes of elevated PHQ-9 (≥10), AUDIT (≥ 8), DAST (≥ 3), or sexual stigma score (highest quartile). For each elevated score, participants were assigned one point, summing to a range of 0 (no elevated scores) to 4 (all scores elevated). Models were adjusted for sociodemographic factors and study site. To reduce sparsity for this final analysis, we dichotomized educational attainment and employment status, and combined those participants having 3 or 4 elevated scores. Statistical analyses were conducted using Stata/SE 17.0.

## Results

Distribution of age, employment status, marriage and cohabitation with a female partner were similar (Table 1). TGW had lower educational attainment and were more likely to report living with a male sex partner, recent RAI, and a male partner at last sex; TGW also reported greater numbers of male sex partners and paying male sex partners. TGW were marginally statistically more likely to report having a paying female sex partner, having engaged in group sex, and having paid for sex with cash.

**Table 1:** Distribution of characteristics of transgender women vs. cis-gender men who have sex with men (MSM), Kenya

Characteristics^a^	Transgender women *N* = 108 (12.9%) n (%)	Cis-gender MSM *N* = 730 (87.5%) n (%)	*P*-value^‡^
Site			< 0.001
Kisumu	39 (36.1)	261 (35.5)	
Nairobi	21 (19.4)	277 (37.8)	
Mtwapa	18 (16.7)	112 (15.3)	
Malindi	30 (27.8)	83 (11.4)	
*Socio-Demographics*			
Age group, in years			0.924
18–20	20 (18.5)	141 (19.4)	
21–23	47 (43.5)	294 (40.4)	
24–26	29 (26.9)	199 (27.4)	
27–29	12 (11.1)	93 (12.8)	
Median (IQR) age in years	23 (21–25)	23 (21–25)	0.782
Highest educational attainment			< 0.001
Primary 1–8	33 (30.6)	110 (15.1)	
Some secondary	23 (21.3)	88 (12.1)	
Secondary	32 (29.6)	355 (48.6)	
Some college or more	20 (18.5)	177 (24.2)	
Employment status			0.354
Unemployed	40 (37.0)	293 (40.7)	
Self-Employed/casual work	50 (46.3)	282 (39.2)	
Employed, parttime or full time	18 (16.7)	145 (20.1)	
Ever married to female	12 (11.1)	92 (12.6)	0.661
Currently living with female wife or female sex partner	16 (14.8)	113 (15.5)	0.854
Currently living with male sex partner	61 (56.5)	332 (45.5)	0.034
*Sexual practices (recall period is past 3 months unless otherwise specified)*			
Receptive anal intercourse (RAI) with a male partner	93 (86.1)	410 (56.6)	< 0.001
Used condom last RAI	72 (77.4)	333 (80.4)	0.512
Median (IQR) number of total male sex partners	4 (2–7)	3 (2–6)	0.005
0-1^b^	6 (5.6)	115 (15.9)	0.012
2	24 (22.4)	150 (20.8)	
3	14 (13.1)	124 (17.2)	
4 or more	63 (58.9)	334 (46.2)	
Median (IQR) number of paying male sex partners	2 (1–4)	1 (0–3)	0.021
Zero	25 (23.8)	209 (29.2)	0.008
1–2	36 (34.3)	313 (43.7)	
3 or more	44 (41.9)	194 (27.1)	
Median (IQR) number of female sex partners	1 (0–2)	1 (0–3)	0.488
Zero	45 (44.5)	311 (44.1)	0.518
1–2	34 (33.7)	207 (29.4)	
3 or more	21 (21.8)	187 (26.5)	
Any paying female sex partners	37 (36.3)	193 (27.9)	0.080
Ever in lifetime had sex with a transgender female	74 (68.5)	350 (48.0)	< 0.001
Last sex partner was male	105 (97.2)	620 (85.1)	0.001
Used condom at last sex	76 (72.4)	485 (78.2)	0.186
Lubricant used at last sex	94 (89.5)	513 (82.9)	0.087
Paid for sex with cash	49 (45.8)	266 (36.5)	0.065
Engaged in group sex	28 (25.9)	134 (18.4)	0.065
Any interpersonal violence	27 (25.0)	154 (21.1)	0.361
Any childhood abuse	76 (71.0)	441 (60.8)	0.042
*Mental health, stigma, and substance use*			
PHQ-9, median (IQR)	5 (1–8)	3 (1–7)	0.112
PHQ-9 ≥ 10 (moderate/severe depressive symptoms)	21 (19.8)	96 (13.3)	0.071
Stigma and discrimination score, median (IQR)	9 (4–15)	7 (3–12)	0.015
AUDIT, median (IQR)	7 (1–12)	4 (0–10)	0.066
AUDIT ≥ 8 Hazardous/Harmful alcohol use	44 (41.5)	250 (34.9)	0.183
DAST, median (IQR)	0 (0–3.75)	0 (0–4)	0.588
DAST > 3 (moderate/substantial/severe)	31 (28.7)	244 (33.6)	0.316
Number of elevated scores for mental health (PHQ ≥ 10), AUDIT (≥ 8), DAST (≥ 3), and stigma (highest quartile)			0.474
0	34 (32.7)	278 (39.8)	
1	36 (34.6)	202 (28.9)	
2	18 (17.3)	127 (18.2)	
3	11 (10.6)	72 (10.3)	
4	5 (4.8)	19 (2.7)	
*HIV Risk and Prevention*			
What are your chances of getting HIV/AIDS			0.030
No chance at all	31 (28.7)	309 (42.5)	
Small chance	38 (35.2)	212 (29.1)	
Moderate chance	14 (13.0)	95 (13.1)	
Great chance	25 (23.2)	112 (15.4)	
Ever taken PrEP			0.038
Never heard of it	10 (9.3)	107 (14.7)	
No	40 (37.0)	322 (44.2)	
Yes	58 (53.7)	300 (41.1)	
Still taking PrEP (among those ever taken PrEP)	45 (77.6)	183 (61.0)	0.016
*Gender Affirming Therapies (GAT) Taken or Currently Using*			
Any feminizing GAT	15 (14.0)	59 (8.2)	0.048
Feminizing hormonal agents such as estradiol or progesterone	12 (11.2)	32 (4.4)	0.004
Anti-androgen agents such as aldactone or spironolactone	3 (2.8)	21 (2.9)	> 0.999
Silicone breast implants	2 (1.9)	12 (1.7)	0.700

^a^Not all cells sum to N due to missing data

^b^Includes *n* = 19 participants who reported zero male sex partners in the past 3 months. The number of male sex partners is not inclusive of the number of paying male sex partners, as these questions were assessed separately

^‡^Chi-square test applied for categorical variables; Wilcoxon rank sum test applied for continuous data comparisons, as all had non-normal distribution

**Fig. 1:**
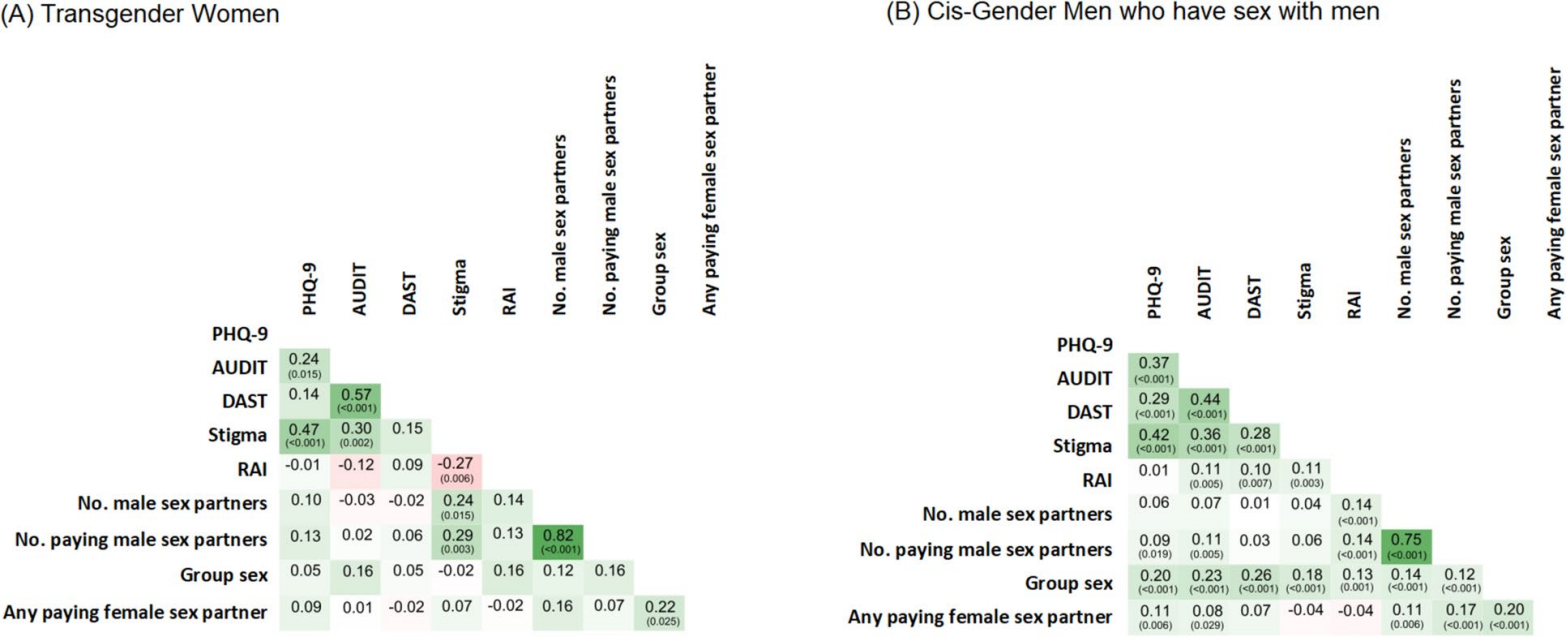
Panels show heat plots of the correlations of psychometric scales and sexual practices among (**A**) Transgender Women and (**B**) Cis-Gender Men who have sex with men. In both plots, green colors represent stronger positive correlation, and red colors represent negative correlations, with intensity of colors representing strength of correlation. Within each cell, correlation coefficients are reported with p-values in parenthesis underneath the correlation value

Summary scores and dichotomized values of psychometric scales are presented in Table 1; specific item responses are presented in Supplemental Table 4. TGW reported significantly higher prevalence of childhood abuse and greater sexual stigma than cis-MSM. Elevated PHQ (≥10) was marginally more frequent for TGW than cis-MSM (19.8% vs. 13.3%, *p*=0.071), though they did not differ from cis-MSM on hazardous/harmful alcohol or harmful drug use or on occurrence of interpersonal violence. Sexual stigma, PHQ-9, AUDIT, and DAST scores were positively correlated with one another (Fig. 1). Notably, RAI was statistically significantly inversely associated with stigma among TGW (rho = -0.27, *p*=0.006), and was positively correlated with stigma among cis-MSM (rho = 0.11, *p*=0.003). Number of male sex partners and paying male sex partners were significantly positively correlated with stigma among TGW, but not among cis-MSM.

### Factors associated with greater number of elevated mental health, sexual stigma, alcohol and drug use scores

Overall, 38.7% of participants did not have any elevated scores. The frequency distribution of the number of adverse outcomes scores did not differ between TGW and cis-MSM (*p*=0.474, Table 1). However, in multivariable modeling adjusted for age, educational attainment, employment, and study site, TGW were more likely than cis-MSM to have an increased number of elevated mental health, stigma, and substance use outcomes (Table 2). Specifically, compared to cis-MSM, TGW had a 1.24-fold greater prevalence ratio for having two adverse outcomes and a 1.25-fold greater prevalence ratio for having three or four adverse outcomes. Of note, older age was associated with having three or four adverse outcomes (aPR 1.18, 95% CI 1.04 – 1.33). There was inconsistent association with study site and increasing number of elevated scores.

**Table 2:** Results of multivariable multivariate regression: association of Transgender Women (TGW) vs. Cis-gender men who have sex with men (Cis-MSM) in relation to number of overlapping elevated scores for depressive symptoms, alcohol and drug use, and stigma score in the highest quartile

Variables	**One elevated score vs. No elevated scores** **aPR (95% CI), *p*-value**	**Two elevated scores vs. No elevated scores** **aPR (95% CI), *p*-value**	**Three or four elevated scores vs. No elevated scores** **aPR (95% CI), *p*-value**
TGW vs. cis-MSM	1.47 (0.98 – 2.21), 0.062	1.24 (1.25 – 1.71), < 0.001	1.25 (1.15 – 1.46), < 0.001
Age in years, continuous	1.07 (1.03 – 1.12), 0.001	1.03 (0.92 – 1.16), 0.589	1.18 (1.04 – 1.33), 0.010
Highest education completed: Secondary or more vs. less than Secondary school	1.16 (0.64 – 2.10), 0.620	1.30 (0.75 – 2.24), 0.354	0.73 (0.40 – 1.32), 0.297
Unemployed vs. Employed	0.66 (0.37 – 1.18), 0.166	0.58 (0.47- 0.72), < 0.001	0.80 (0.63 – 1.03), 0.081
Study Site			
Kisumu	reference	reference	reference
Nairobi	1.48 (1.31 – 1.68), < 0.001	0.78 (0.68 – 0.90), < 0.001	1.50 (1.30 – 1.73), < 0.001
Mtwapa	0.83 (0.78 – 0.88), < 0.001	0.45 (0.44 – 0.46), < 0.001	0.91 (0.83 – 0.99), 0.028
Malindi	2.06 (1.66 – 2.55), < 0.001	0.28 (0.57 – 0.80), < 0.001	2.16 (1.72 – 2.71), < 0.001

*aPR* Adjusted Prevalence Ratio, *95% CI* 95% Confidence Interval

### Results of multivariable modeling: persistent differences between Cis-MSM and TGW

Differences in sexual practices reported by TGW vs. cis-MSM persisted after controlling for age, educational attainment, employment status, and study site (Table 3). For the past 3-month recall period, TGW were more likely to report: RAI (adjusted prevalence ratio [aPR] = 1.59, 95% CI: 1.32 – 1.92), engaging in group sex (aPR=1.15, 95% CI: 1.04 – 1.27), greater number of male sex partners (e.g., aPR for 4 or more sex partners =3.31, 95% CI: 2.52 – 4.35), and more paying male sex partners (aPR for 3 or more paying sex partners = 1.58, 95% CI: 1.04 – 2.39). Compared to cis-MSM, TGW were more likely to report moderate to severe depressive symptoms (aPR=1.42, 95% CI: 1.01 – 1.55) and had on average marginally higher stigma score (2.47 points, *p*=0.072), while TGW and cis-MSM had similar alcohol and drug abuse scores.

**Table 3:** Results of crude and multivariable regression models: association of transgender women vs. cis-gender men who have sex with men in relation to outcomes of sexual practices, stigma, hazardous alcohol drinking, and depressive symptoms

Variables^a^	Crude Prevalence Ratio (95% CI), *p*-value	Adjusted^b^ Prevalence Ratio (95% CI), *p*-value
Any receptive intercourse, yes vs. no	1.52 (1.37 – 1.69), < 0.001	1.59 (1.32 – 1.92), < 0.001
Number of male sex partners		
0–1	reference	reference
2	3.07 (2.04 – 4.60), < 0.001	3.08 (1.94 – 4.90), < 0.001
3	2.16 (0.98 – 4.77), 0.052	2.13 (0.82 – 5.54), 0.122
4 or more	3.62 (2.72 – 4.81), < 0.001	3.31 (2.52 – 4.35), < 0.001
Number of paying male sex partners		
Zero	reference	reference
1–2	0.96 (0.58 – 1.59), 0.879	0.81 (0.53 – 1.24), 0.331
3 or more	1.90 (1.04 – 3.46), 0.037	1.58 (1.04 – 2.39), 0.031
Any paying female sex partner, yes vs. no	1.30 (0.76 – 2.24), 0.342	0.82 (0.41 – 1.63), 0.577
Any group sex, yes vs. no	1.41 (1.29 – 1.54), < 0.001	1.15 (1.04 – 1.27), 0.006
Depressive symptoms moderate to severe: PHQ-9 dichotomized at 10	1.49 (1.31 – 1.70), < 0.001	1.42 (1.25– 1.62), < 0.001
Hazardous/Harmful alcohol use: AUDIT score dichotomized at 8	1.19 (0.91 – 1.55), 0.196	1.10 (0.91 – 1.33), 0.301
Moderate/Substantial/Severe substance use: DAST score dichotomized at 3	0.86 (0.60 – 1.22), 0.387	0.84 (0.62 – 1.16), 0.292
***Linear Regression: Continuous Outcomes***	**Crude Coefficient (95% CI), *p*-value**	**Adjusted Coefficient (95% CI), *p*-value**
Stigma Score	2.04 (-0.59 – 4.66), 0.091	2.47 (-0.41 – 5.36), 0.072
Depressive symptoms	0.79 (0.04 – 1.54), 0.044	0.60 (0.01–1.19), 0.048
AUDIT score	1.05 (-1.28 – 3.38), 0.248	0.87 (-1.16 – 2.89), 0.267
DAST score	-0.17 (-0.92 – 0.58), 0.526	-0.18 (-0.66 – 0.30), 0.320

^a^Recall periods for all sexual behaviors are “past 3 months”

^b^Adjusted for: age, educational attainment, employment status, site

As a sensitivity analysis, we classified participants who reported being male and taking GAT as TGW. The GAT assessed were hormones with feminizing effect, anti-androgen agents, and silicone implants (Table 1), and were reported by 14% of TGW and 8.2% of cis-MSM (*p*=0.048). When comparing male-identifying individuals taking GAT with cis-MSM, the results of modeling produced similar results as comparisons of TGW with cis-MSM (Supplemental Table 2). Compared to cis-MSM, male-identifying individuals taking GAT had elevated depressive symptoms, potentially harmful alcohol use, and elevated sexual stigma, but did not differ in harmful substance use. Compared to cis-MSM, male-identifying individuals taking GAT had an increased likelihood of reporting RAI and group sex, greater number of male sex partners and paying male sex partners, similar to TGW, and were more likely to report having a paying female sex partner (aPR=1.64, 95% CI: 0.93 – 2.91), in contrast to TGW.

## Discussion

In keeping with results from numerous studies globally, compared to cis-MSM, TGW in our study had increased measures of: sexual behavioral risk [27, 28], stigma and discrimination [27, 28], depressive symptoms [29–31], and potentially harmful alcohol use [30, 31]. Greater sexual risk taking among TGW as compared to cis-MSM has been associated with unemployment and lack of or limited income and financial support, stemming from discrimination, stigma, and other structural factors that disproportionately affect TGW more than cis-MSM, such as poorer access to health care and less economic opportunity [27]. Additionally, increased sexual risk-taking and poorer health states among TGW may be driven by the negative effects of gender dysphoria uniquely felt by trans persons [32, 33]. In resource-rich settings, engagement (linkage, utilization, adherence) with mental health, alcohol or substance use treatment has been low for trans and gender non-conforming individuals [29], and has been adversely impacted by negative health care experiences, gender-invalidation, -avoidance, -generalizing, and -pathologizing, emphasizing the importance of culturally competent care [29, 34]. These challenges are likely increased in resource-limited settings.

The exclusion of MSM, TGW, and other sexual and gender minorities from policy may present another challenge to recognizing and affirming access to mental health, sexual, and reproductive health care. As others have recommended [16], the Kenya Mental Health Policy 2015-2030 [35] should explicitly address sexual and gender minority persons, to ensure they receive necessary care, install training programs, and work with and empower civil service organizations (CSOs). In an analysis of legislative and policy instruments developed and adopted by the African Union, Izugbara et al. find that although the documents affirm the rights to sexual and reproductive health, socioeconomic opportunities, and freedom from discrimination and criminalization, these policies do not specifically discuss sexual and gender minority persons [36]. This represents a missed opportunity to strengthen policy and programmatic opportunities and advance sexual and gender minority rights.

In a 2017 cross-sectional study in Nairobi of 70 TGW and 592 cis-MSM using respondent-driven sampling to increase representativeness [37], HIV prevalence was 41% among TGW compared to 25% among cis-MSM. Similar to our current findings from three different Kenyan sites, TGW in the Nairobi study were more likely than cis-MSM to report RAI, transactional sex with men, and higher number of recent male sex partners; in the Nairobi study TGW also reported higher frequency of condomless RAI, which we did not observe. Because our cohort enrolled HIV-uninfected participants aged 18-29 years (23% of the Nairobi study sample were aged ≥30 years), our results are not directly comparable, but together these two studies show consistency over time and across differing methodologies in elevated sexual vulnerabilities for TGW in Kenya. Follow-up of participants in the Tatu Pamoja cohort completed in December 2022, and factors associated with STI and HIV incidence will be fully explored, including concordance of risk perception with sexual practices, and how this may differ for TGW vs. cis-MSM.

In a multinational study, transgender and non-binary persons who do not have access to gender-affirming resources were more likely to have increased prevalence of depressive symptoms, anxiety, and suicidal ideation [38]. Our analysis also showed that TGW were more likely than cis-MSM to have greater overlap of elevated depressive symptoms, drug and alcohol use, and stigma. Thus, enhancing TGW health outcomes will require attention to their unique gender-identity needs and the multiple, simultaneously occurring health concerns of stigma, depressive symptoms, alcohol and drug use, and sexual risk. Optimizing HIV prevention and care, including closing the gap in PrEP awareness and use, will need understanding of sexual practices and drivers of this, appreciation for local cultural context, and thorough identification and evaluation of referrals, ensuring linkages are accessible and effective [39]. The HIV differentiated service delivery framework for key populations highlights differences between TGW and cis-MSM, such as training and expertise in transition care [40], and our findings contribute to understanding on differences in factors that may support optimized and differentiated HIV service delivery.

In our study, a substantial number of participants who reported their current gender as male reported using feminizing GAT and characteristics of these participants were largely similar to characteristics of TGW participants, and results of modeling were similar. However, dichotomizing gender as cis-MSM or TGW harbors inherent misclassification that could result in misunderstanding individuals’ needs. There are limited data in Kenya and sub-Saharan Africa on processes of gender development and gender-related sense of self, gender perception, gender expression, gender presentation, and gender identity and labels; such assessment would inform gender-affirming interventions [41]. Among 300 MSM participants in a Tanzanian study, 17% identified as “transsexual or transgender”, and among those, 70% identified themselves as a “woman” [42]. This study further identified that time spent “living as a woman” was variable among participants identifying as transsexual or transgender, and authors acknowledged that varying concepts of the transfeminine identity may have led to misclassification. As explained by M’Baye, “studying transgenderism and homosexuality in African contexts is challenging” due to reliance on concepts that are primarily Western-derived [43]. Generating contextually centered knowledge on gender-related identities and experiences could lead to measures that better represent the health and well-being of trans- and gender non-conforming communities, and lead to development of interventions that better serve their needs.

Our assessment of GAT was not comprehensive, did not differentiate between ever or current GAT use, and we did not verify actual use of GAT. It is possible there was agreement bias or that the questions may not have been understood, especially as GAT in Kenya are relatively recent and not widely available. Framing therapies as “gender-affirming” may have biased participant responses, with the wording perhaps implying seeking a different gender. A qualitative study by Kimani et al. among TGW in coastal Kenya highlighted a desire for GAT as a priority service [44]. In addition to hormone therapy, surgery (e.g., vaginoplasty, facial reconstruction, breast augmentation), or other gender-affirming medications, a comprehensive range of GAT may include gynecologic and urologic care, emotional support, mental health counseling and therapy, other body modifiers (e.g., binders), voice and communication therapy, hair removal, and cosmetic supplies and services [45]. As part of developing effective differentiated HIV prevention and care for trans persons in Kenya, there is need to assess the current status of gender-affirming care in terms of what is available in the public and private sectors, how accessible such services are, and how they can be improved.

### Limitations

A limitation of this study is that in addition to having insufficient methods to capture the range of TGW identity, we did not have metrics for non-binary categorization and excluded participants who did not identify as either male or female. We based the classification of TGW on two variables that were in conflict in a few instances, and we defaulted to TGW classification if “female” or “woman” was reported in response to either question, though there could have been data entry errors during ACASI use. We measured educational attainment and employment status, but did not have comprehensive measures including housing stability, food security, income, or financial dependency, and which may have provided additional insight towards differences between TGW and cis-MSM. The sexual stigma scale was not tailored to TGW and therefore does not reflect sexual stigma experienced differently by cis-MSM and TGW. In general, our survey instrument was not designed to assess the experience of TGW; future survey rounds will incorporate TGW-specific measures, including piloting a transgender stigma scale and assessment of trans-specific gender developmental milestones. This study comprised a large sample of participants from across three urban and peri-urban areas of Kenya. However, self-selection to participate may affect generalizability, and individuals who were outside of the eligibility criteria (e.g., men of younger or older age or those living with HIV) are not represented. Results may not generalize to gender and sexual minorities living in rural areas. The participants in our study had been previously engaged with these organizations for programmatic services and research studies, which may have affected their reporting and sexual practices. As with any study, data are self-reported and subject to social desirability and other reporting biases, though ACASI may have mitigated this and there were minimal missing data and largely consistent associations across study sites.

## Conclusions

In our study across three major metropolitan areas in Kenya, TGW were more likely than cis-MSM to report depressive symptoms, harmful or hazardous alcohol use, greater perceived and experienced stigma, and increased sexual risk taking, independent of sociodemographic factors. We also identified a need for research on emerging gender identities and the complex dimensions of this, which may be a barrier to effective health evaluation and intervention development for transgender and gender non-conforming persons. Nevertheless, our analysis affirms the need for multiple programmatic interventions specific to transgender populations.

### Supplementary Information

**Additional file 1: Supplemental Table 1.** Distribution of characteristics of Transgender women and men who report taking feminizing gender affirming treatments (GAT) compared to Cis-gender men who have sex with men (Cis-MSM), Kenya. **Supplemental Table 2.** Results of Crude and Multivariable Regressions: Association of Transgender Women (TGW) or Men Taking Feminizing Gender Affirmative Therapy (GAT) Compared to Cis-Gender Men who have Sex with Men (Cis-MSM) in Relation to: Sexual Practices, Stigma, Drug and Alcohol Use, and Depressive Symptoms. **Supplemental Table 3.** Scales used: performance, alterations. **Supplemental Table 4.** Distribution individual items from scales of depressive symptoms, alcohol use, drug use, and interpersonal violence, compared between Transgender women and Cis-gender men who have sex with men (Cis-MSM), Kenya.

## Data Availability

The data that support the findings of this study are available from the corresponding author, SDM, upon reasonable request and approval of principal investigators.
