# Supplementary material for: Transgender women in Kenya experience greater stigma, depressive symptoms, alcohol and drug use and risky sexual practices than cis-gendered men who have sex with men
Source: BMC Public Health. 2023 Aug 5;23:1493. doi: 10.1186/s12889-023-16348-6 (PMC10403860; doi:10.1186/s12889-023-16348-6)
Supplement: Supplementary file 1 — Additional file 1: Supplemental Table 1. Distribution of characteristics of Transgender women and men who report taking feminizing gender affirming treatments (GAT) compared to Cis-gender men who have sex with men (Cis-MSM), Kenya. Supplemental Table 2. Results of Crude and Multivariable Regressions: Association of Transgender Women (TGW) or Men Taking Feminizing Gender Affirmative Therapy (GAT) Compared to Cis-Gender Men who have Sex with Men (Cis-MSM) in Relation to: Sexual Practices, Stigma, Drug and Alcohol Use, and Depressive Symptoms. Supplemental Table 3. Scales used: performance, alterations. Supplemental Table 4. Distribution individual items from scales of depressive symptoms, alcohol use, drug use, and interpersonal violence, compared between Transgender women and Cis-gender men who have sex with men (Cis-MSM), Kenya. [file 12889_2023_16348_MOESM1_ESM.docx]

**Supplemental Table 1. Distribution of characteristics of Transgender women and men who report taking feminizing gender affirming treatments (GAT) compared to Cis-gender men who have sex with men (Cis-MSM), Kenya**

| Characteristics^†^ | Transgender women and men who report taking GAT  N=167 (19.9%)  n (%) | Cis-MSM  N=671 (80.1%)  n (%) | P-value‡ |
| --- | --- | --- | --- |
| Site  Kisumu  Nairobi  Mtwapa  Malindi | 66 (39.5)  43 (25.7)  22 (13.2)  36 (21.6) | 232 (34.6)  254 (37.9)  108 (16.1)  77 (11.5) | 0.001 |
| *Socio-Demographics* |  |  |  |
| Age group, in years  18-20  21-23  24-26  27-29 | 31 (18.6)  70 (41.9)  45 (26.9)  21 (12.6) | 130 (19.5)  271 (40.6)  183 (27.4)  84 (12.6) | 0.989 |
| Median (IQR) age in years | 23 (21-25) | 23 (21-25) | 0.881 |
| Highest educational attainment (educreco1)  Primary 1-8  Some secondary  Secondary  Some college or more | 44 (26.4)  37 (22.2)  55 (32.9)  31 (18.6) | 99 (14.8)  74 (11.0)  332 (49.5)  166 (24.8) | <0.001 |
| Employment status (employ)  Unemployed  Self-Employed/casual work  Employed, parttime or full time | 60 (36.6)  73 (44.5)  31 (18.9) | 273 (41.1)  259 (39.0)  132 (19.9) | 0.421 |
| Ever married to female | 22 (13.2) | 82 (12.2) | 0.743 |
| Currently living with female wife or female sex partner | 28 (17.1) | 101 (15.0) | 0.511 |
| Currently living with male sex partner | 101 (60.5) | 292 (43.6) | <0.001 |
| *Sexual practices (recall period is past 3 months unless otherwise specified)* |  |  |  |
| Had receptive anal intercourse (RAI) with a male partner | 135 (80.8) | 368 (55.3) | <0.001 |
| Used condom at last RAI with male partner | 107 (79.3) | 298 (80.1) | 0.833 |
| Median (IQR) number of male sex partners | 4 (2-7) | 3 (2-6) | <0.001 |
| Median (IQR) number of paying male sex partners | 2 (1-4) | 1 (0-3) | 0.009 |
| Any female sex partner | 97 (60.6) | 353 (54.6) | 0.173 |
| Median (IQR) number of female sex partners | 1 (0-2.75) | 1 (0-3) | 0.590 |
| Number of paying female sex partners  None  One  Two  Three or more | 33 (34.7)  27 (28.4)  16 (16.8)  19 (20.0) | 176 (51.2)  79 (23.0)  39 (11.3)  50 (14.4) | 0.039 |
| Ever had sex with a transgender female | 105 (62.9) | 319 (47.6) | <0.001 |
| Last sex partner was male | 158 (95.2) | 567 (84.5) | <0.001 |
| Used condom at last sex with male sex partner | 118 (74.7) | 443 (78.1) | 0.360 |
| Lubricant used at last sex with male sex partner | 138 (87.3) | 469 (82.9) | 0.176 |
| Paid for sex with cash | 79 (47.9) | 236 (35.2) | 0.003 |
| Engaged in group sex | 45 (27.1) | 117 (17.5) | 0.005 |
| *Interpersonal Violence (IPV) past 3 months* |  |  |  |
| Median (IQR) IPV score | 0 (0-1) | 0 (0-0) | 0.068 |
| Any IPV | 44 (26.4) | 137 (20.5) | 0.098 |
| *Mental Health and Substance Use* |  |  |  |
| Median (IQR) PHQ score | 5 (2-8.5) | 3 (1-7) | 0.002 |
| PHQ > 10: Moderate/moderately severe/   severe depressive symptoms | 34 (20.7) | 83 (12.5) | 0.007 |
| Median (IQR) AUDIT score | 6.5 (0.25 – 13) | 4 (0.10) | 0.030 |
| AUDIT > 8: Hazardous/Harmful alcohol   use | 70 (42.7) | 224 (34.0) | 0.038 |
| Median (IQR) DAST score | 0 (0-3) | 0 (0-3) | 0.8589 |
| DAST > 3: Moderate/substantial/severe   substance use | 44 (26.5) | 173 (25.9) | 0.865 |
| Median (IQR) childhood abuse score | 1 (0-2) | 1 (0-2) | 0.083 |
| Any childhood abuse | 111 (66.9) | 406 (61.0) | 0.160 |
| Median (IQR) Stigma and discrimination score | 8.5 (4-15) | 7 (3-12) | 0.006 |
| *HIV Risk and Prevention* |  |  |  |
| What are your chances of getting HIV/AIDS  No chance at all  Small chance  Moderate chance  Great chance | 53 (31.7)  58 (34.7)  20 (12.0)  36 (21.6) | 287 (42.9)  192 (28.7)  89 (13.3)  101 (15.1) | 0.026 |
| Ever taken PrEP | 88 (60.3) | 270 (47.0) | 0.004 |
| Still taking PrEP (among those   ever taken PrEP) | 71 (80.7) | 157 (58.2) | <0.001 |

† Not all cells sum to N due to missing data.

‡ Chi-square test applied for categorical variables; Fisher’s exact employed where cell size n<5; Wilcoxon rank sum test applied for continuous data comparisons, as all had non-normal distribution

**Supplemental Table 2. Results of Crude and Multivariable Regressions: Association of Transgender Women (TGW) or Men Taking Feminizing Gender Affirmative Therapy (GAT) Compared to Cis-Gender Men who have Sex with Men (Cis-MSM) in Relation to: Sexual Practices, Stigma, Drug and Alcohol Use, and Depressive Symptoms.**

| **Variables**† | **Crude PR (95% CI), p-value** | | **Adjusted**‡ **PR (95% CI), p-value** | |
| --- | --- | --- | --- | --- |
|  | **TGW vs.**  **Cis-MSM** | **Men taking GAT vs. Cis-MSM** | **TGW vs.**  **Cis-MSM** | **Men taking GAT vs. Cis-MSM** |
| Any receptive intercourse, yes vs. no§ | 1.56 (1.40 – 1.73), <0.001 | 1.29 (1.16 – 1.43), <0.001 | 1.63 (1.35 – 1.97), <0.001 | 1.28 (1.14 – 1.44), <0.001 |
| Number of male sex partners  0-1  2  3  4 or more | Reference  3.19 (2.17 – 5.08), <0.001  2.27 (1.09 – 4.72), 0.028  3.87 (2.89 – 5.19), <0.001 | Reference  4.15 (1.61 – 10.7), 0.003  2.92 (0.54 – 16.0),  0.216  3.68 (1.83 – 7.43), <0.001 | Reference  3.42 (2.13 – 5.49), <0.001  2.26 (0.92 – 5.56), 0.075  3.65 (2.83 – 4.71), <0.002 | Reference  6.50 (1.13 – 37.4), 0.036  4.11 (0.33 – 51.2), 0.272  6.16 (1.18 – 32.3), 0.031 |
| Number of paying male sex partners  Zero  1-2  3 or more | Reference  1.00 (0.62 – 1.61), 0.995  2.05 (1.14 – 3.69), 0.017 | Reference  1.80 (1.13 – 2.87), 0.013  2.68 (1.98 – 3.62), <0.001 | Reference  0.84 (0.55 – 1.27), 0.405  1.73 (1.14 – 2.61),  0.010 | Reference  1.64 (1.23 – 2.18), 0.001  2.86 (1.95 – 4.19), <0.001 |
| Any paying female sex partner, yes vs. no^3^ | 1.38 (0.73 – 2.58), 0.320 | 1.69 (0.91 – 3.15), 0.097 | 1.01 (0.49 – 2.07), 0.983 | 1.64 (0.93 – 2.91), 0.089 |
| Any group sex, yes vs. no | 1.49 (1.32 – 1.67), <0.001 | 1.68 (1.34 – 2.10), <0.001 | 1.20 (1.11 – 1.30), <0.001 | 1.55 (1.03 – 2.32), 0.035 |
| Depressive symptoms moderate to severe: PHQ-9 dichotomized at 10 | 1.59 (1.39 – 1.81),  <0.001 | 1.80 (1.11 – 2.91),  0.018 | 1.50 (1.26 – 1.80),  <0.001 | 1.67 (1.04 – 2.68),  0.033 |
| Hazardous/Harmful alcohol use: AUDIT score dichotomized at 8 | 1.22 (0.92 – 1.63),  0.171 | 1.32 (0.92 – 1.88),  0.129 | 1.13 (0.91 – 1.40),  0.285 | 1.21 (0.73 – 2.00), 0.460 |
| Moderate/Substantial/Severe substance use: DAST score dichotomized at 3 | 0.86 (0.60 – 1.24), 0.429 | 1.14 (0.70 – 1.87), 0.593 | 0.85 (0.62 – 1.17), 0.321 | 1.11 (0.72 – 1.72), 0.626 |
| ***Linear Regression: Continuous Outcomes*** |  |  |  |  |
| Stigma Score, continuous | 2.22 (-0.34 – 4.79), 0.070 | 2.32 (-0.34 – 4.97), 0.069 | 2.65 (-0.27 – 5.57), 0.063 | 2.04 (-0.46 – 4.55), 0.081 |
| Depressive symptoms, continuous | 0.94 (0.32 – 1.56),  0.017 | 1.88 (-0.22 – 3.98),  0.065 | 0.75 (0.32 – 1.18),  0.011 | 1.81 (0.03 – 3.49),  0.048 |
| AUDIT score | 1.22 (-1.39 – 3.78), 0.229 | 2.06 (-1.34 – 5.46), 0.149 | 0.99 (-1.37 – 3.35), 0.274 | 1.44 (-3.19 – 6.08), 0.395 |
| DAST score | -0.11 (-0.89 – 0.67), 0.695 | 0.80 (-1.17 – 2.77), 0.288 | -0.12 (-0.62- 0.37), 0.483 | 0.64 (-0.90 – 2.17), 0.279 |

† All recall periods for sexual behaviors are “past 3 months”

‡ Adjusted for: age, educational attainment, employment status, site

§ Negative binomial regression fitted because log binomial did not converge

**Supplemental Table 3. Scales used: performance, alterations.**

| **Scale [refs]** | **Cronbach’s alpha** | **Alterations** |
| --- | --- | --- |
| PHQ-9 [18] | 0.82  In our previous study in Kisumu [20], PHQ-9 performed with Cronbach’s alpha = 0.82. In a previous study among MSM in Mombasa [24], Cronbach’s alpha of PHQ-9 was 0.86. | - The response categories and recall are verbatim to the original PHQ-9. - The original questions are framed as “Over the last 2 weeks, how often have you been bothered by any of the following problems?” We worded these to the problem itself, rather than “bothered by”, which was felt to be confusing. For example, “In the last 2 weeks, how often have you been bothered by poor appetite or overeating?” was changed to “In the last 2 weeks, how often have you had poor appetite or have overeaten”. As a second example, “How often have you been bothered by feeling down, depressed, or hopeless” was changed to “How often have you felt down, depressed, or hopeless?” Other questions were changed similarly. |
| AUDIT [19] | 0.84  Previously in Kisumu [20], AUDIT performed with Cronbach’s alpha = 0.91. Previously in Mombasa [24], Cronbach’s alpha of AUDIT was 0.87. | Two questions were altered to clarify the recall period in advance of expected responses.   - The original question “Have you or someone else ever been injured as a result of your drinking?” (response categories: No; Yes, but not in the last year; Yes, during the last year) was changed to “Have you or someone else ever been injured as a result of your drinking, either in the past or more recently?” (response categories as above) - The question “Has a relative or friend or a doctor or another health worker been concerned about your drinking or suggested you cut down?” (response categories: No; Yes, but not in the last year; Yes, during the last year) was changed to “Has a relative or friend, or a doctor or other health worker ever been concerned about your drinking or suggested you cut down, either in the past or more recently?” (response categories as above). |
| DAST [20-22] | 0.89  Previously in Kisumu [20], DAST performed with Cronbach’s alpha = 0.85. Previously in Mombasa [24], Cronbach’s alpha of DAST was 0.78. | - The introduction to the DAST was changed to be specific to the Kenyan context, and read as follows: “The following questions concern information about your potential involvement with drugs excluding alcohol and tobacco during the past 12 months. Some of the drugs we are asking about are miraa (khat), bhang (marijuana), hashish, inhalants (glue, paint), heroin, cocaine, pain medications, sleeping medications, and others. Remember that the questions do not include alcohol or tobacco.” Compared to the original wording: This list removes solvents, tranquilizers (e.g., Valium), stimulants (e.g., speed), hallucinogens (e.g., LSD), and barbiturates; does not refer to marijuana as “cannabis”; does not refer to heroin as “narcotics”, and adds inhalants, pain medications, and sleeping medications. - For each DAST question administered, we prefaced it with “In the past year”. - The question “Do you abuse more than one drug at a time?”, was changed to “In the past year, have you taken more than one drug at a time?” to remove the word “abuse” as it may elicit response bias. - The question “Are you always able to stop using drugs when you want to?”, was changed to “In the past year, have you always been able to stop using drugs when you want to?”, to maintain consistent verb tense. - The question “Do you ever feel bad or guilty about your drug use?”, was changed to “In the past year, have you ever felt bad or guilty about your drug use?” to maintain consistent verb tense. - The question “Does your spouse (or parents) ever complain about your involvement with drugs?” was changed to “In the past year, has your spouse (or parent) ever complained about your involvement with drugs?” to maintain consistent verb tense. |
| CECA [23] | 0.65  Previously in Kisumu [20], CECA performed with Cronbach’s alpha = 0.63. Previously in Mombasa [24], Cronbach’s alpha of DAST was 0.70. | No alterations |
| MSM Stigma Scale [24-25] | 0.84  Previously in Mombasa [24], Cronbach’s alpha of sexual stigma scale was 0.85. | Throughout the most of the question series, the word “homosexual” or “homosexuality” was changed to “sex with men”, to limit potential response bias. For example, the question “How often have you felt that your homosexuality hurt and embarrassed your family?” was changed to “How often have you felt that you hurt or embarrassed your family because you have sex with men?” The question “How often have you been made fun of or called names for being homosexual?” was changed to “How often have you been made fun of or called names because you have sex with men?” Other questions were changed similarly, except for the first question (“How often have you heard that homosexuals are not normal?”) and the fifth question (How often have you had to pretend that you are not homosexual in order to be accepted?”) in the series, which maintained original wording. Response categories followed the original question wording. |

**Supplemental Table 4. Distribution individual items from scales of depressive symptoms, alcohol use, drug use, and interpersonal violence, compared between Transgender women and Cis-gender men who have sex with men (Cis-MSM), Kenya.**

| **Characteristics**† | **Transgender Women N=108 (12.9%)**  **n (%)** | **Cis-gender MSM**  **N=730 (87.1%)**  **n (%)** | **P-value**‡ |
| --- | --- | --- | --- |
| **Patient Health Questionnaire (PHQ-9)**  *Over the last two weeks, how often have you…* |  |  |  |
| Experienced little interest or pleasure in doing things  Not at all  Several days  More than half the days  Nearly all the days | 42 (39.3)  55 (51.4)  5 (4.7)  5 (4.7) | 345 (47.5)  286 (39.3)  38 (5.2)  58 (8.0) | 0.108 |
| Felt down, depressed, or hopeless  Not at all  Several days  More than half the days  Nearly all the days | 57 (52.8)  37 (34.3)  7 (6.5)  7 (6.5) | 405 (55.6)  236 (32.4)  57 (7.8)  31 (4.2) | 0.686 |
| Had trouble falling or staying asleep or sleeping too much  Not at all  Several days  More than half the days  Nearly all the days | 64 (59.3)  33 (30.6)  7 (6.5)  4 (3.7) | 433 (59.4)  215 (29.5)  55 (7.5)  26 (3.6) | 0.980 |
| Felt tired or had little energy  Not at all  Several days  More than half the days  Nearly all the days | 52 (48.2)  43 (39.8)  9 (8.3)  4 (3.7) | 393 (53.8)  253 (34.7)  59 (8.1)  25 (3.4) | 0.693 |
| Had poor appetite or have over eaten  Not at all  Several days  More than half the days  Nearly all the days | 65 (60.2)  33 (30.6)  5 (4.6)  5 (4.6) | 462 (63.6)  198(27.2)  39 (5.4)  30 (4.1) | 0.874 |
| Felt bad about yourself, or that you are a failure or have let yourself of your family down  Not at all  Several days  More than half the days  Nearly all the days | 67 (62.0)  21 (19.4)  10 (9.3)  10 (9.3) | 476 (65.4)  170 (23.3)  43 (5.9)  39 (5.4) | 0.179 |
| Had trouble concentrating on things  Not at all  Several days  More than half the days  Nearly all the days | 62 (57.4)  27 (25.0)  10 (9.3)  9 (8.3) | 483 (66.4)  155 (21.3)  52 (7.1)  38 (5.2) | 0.268 |
| Experienced moving or speaking so slowly other people could have noticed, or being so fidgety or restless that you have been moving around a lot more than usual  Not at all  Several days  More than half the days  Nearly all the days | 71 (66.7)  22 (20.6)  8 (7.5)  6 (5.6) | 526 (72.3)  147 (20.2)  39 (5.4)  16 (2.2) | 0.150 |
| Felt you would be better off dead, or thought of hurting yourself in some way  Not at all  Several days  More than half the days  Nearly all the days | 84 (77.8)  14 (13.0)  5 (4.6)  5 (4.6) | 611 (83.9)  74 (10.2)  20 (2.8)  23 (3.2) | 0.416 |
| **Childhood abuse questionnaire** |  |  |  |
| Ever experienced physical violence as   child or teenager | 67 (62.0) | 387 (53.1) | 0.081 |
| Ever had unwanted sexual experiences as   a child or teenager | 20 (18.5) | 152 (20.2) | 0.567 |
| Ever had upsetting sexual experiences   with related adult or authority figure   before age 17 | 22 (20.6) | 121 (16.7) | 0.309 |
| Ever forced to have sex before age 17 | 26 (24.1) | 119 (16.3) | 0.046 |
| **DAST questionnaire** |  |  |  |
| Used drugs in the past year | 41 (37.9) | 298 (40.8) | 0.572 |
| Taken more than 1 drug at a time in the   past year | 22 (20.4) | 199 (27.3) | 0.234 |
| Able to stop using drugs when you want | 24 (22.2) | 207 (28.4) | 0.178 |
| Blackouts or flashbacks | 19 (17.6) | 138 (18.9) | 0.129 |
| Felt bad or guilty about drug use | 21 (19.4) | 167 (22.9) | 0.726 |
| Complain about involvement with drugs | 22 (20.4) | 158 (21.7) | 0.758 |
| Neglected family because of drug use | 15 (13.9) | 82 (11.3) | 0.424 |
| Engaged in illegal activities | 13 (12.0) | 70 (9.6) | 0.429 |
| Experienced withdrawal symptoms | 17 (15.7) | 102 (13.9) | 0.623 |
| Experienced medical problems | 8 (7.4) | 73 (10.0) | 0.393 |
| **AUDIT questionnaire** |  |  |  |
| How often do you have a drink  containing alcohol  Never  Monthly or less  Two to four times a month  Two to three times per week  Four or more times a week | 32 (29.6)  28 (25.9)  27 (25.0)  16 (14.8)  5 (4.6) | 223 (30.6)  202 (27.7)  164 (22.5)  102 (13.9)  38 (5.21) | 0.973 |
| How many drinks containing alcohol do you have on a typical day when drinking  Never/1-2 drinks  3-4 drinks  5-6 drinks  7-9 drinks  10+ drinks | 75 (69.4)  21 (19.4)  7 (6.5)  3 (2.8)  2 (1.9) | 549 (75.6)  128 (17.6)  35 (4.8)  5 (0.7)  9 (1.2) | 0.170 |
| How often do you have six or more drinks on one occasion  Never  Monthly or less  Two to four times per month  Two to three times per week  Four or more times a week | 63 (58.3)  17 (15.7)  16 (14.8)  9 (8.3)  3 (2.7) | 454 (62.5)  115 (15.8)  90 (12.4)  58 (7.9)  10 (1.4) | 0.666 |
| How often during the last year have you found that you were not able to stop drinking after you had started  Never  Monthly or less  Two to four times per month  Two to three times per week  Four or more times a week | 60 (55.6)  24 (22.2)  13 (12.0)  7 (6.5)  4 (3.7) | 503 (69.0)  116 (15.9)  54 (7.4)  28 (3.8)  28 (3.8) | 0.052 |
| How often during the last year have you failed to do what was normally expected from you because of drinking  Never  Monthly or less  Two to four times per month  Two to three times per week  Four or more times a week | 68 (62.9)  29 (26.9)  5 (4.6)  3 (2.8)  3 (2.8) | 507 (69.7)  157 (21.6)  30 (4.1)  22 (3.0)  11 (1.5) | 0.506 |
| How often during the last year have you need a first drink in the morning to get yourself going after a heavy drinking session  Never  Monthly or less  Two to four times per month  Two to three times per week  Four or more times a week | 78 (72.2)  20 (18.5)  4 (3.7)  3 (2.8)  3 (2.8) | 553 (75.9)  91 (12.5)  27 (3.7)  27 (3.7)  30 (4.1) | 0.540 |
| How often during the last year have you had a feeling of guilt or remorse after drinking  Never  Monthly or less  Two to four times per month  Two to three times per week  Four or more times a week | 72 (66.8)  27 (25.0)  5 (4.6)  1 (0.9)  3 (2.8) | 505 (69.4)  145 (19.9)  29 (3.9)  22 (3.0)  27 (3.7) | 0.587 |
| How often during the last year have you been unable to remember what happened the night before because you had been drinking  Never  Monthly or less  Two to four times per month  Two to three times per week  Four or more times a week | 77 (71.3)  24 (22.2)  1 (0.9)  6 (5.6)  0 (0.0) | 507 (69.5)  153 (20.9)  30 (4.1)  20 (2.7)  19 (2.6) | 0.087 |
| Have you or someone else ever been injured as a result of your drinking, either in the past or more recently  No  Yes, during the last year  Yes, but not in the last year | 64 (60.4)  20 (18.9)  22 (20.8) | 522 (71.7)  107 (14.7)  99 (13.6) | 0.050 |
| Has a relative or friend, or doctor or other health worker ever been concerned about your drinking or suggested you cut down, either in the past or more recently  No  Yes, during the last year  Yes, but not in the last year | 76 (71.0)  19 (17.8)  12 (11.2) | 533 (73.4)  103 (14.2)  90 (12.4) | 0.609 |
| **Interpersonal Violence*:*** *In the past 3 months, have you been in a relationship with a person who…* |  |  |  |
| Threatens, frightens, insults, or   treats you badly? | 12 (11.1) | 83 (11.4) | 0.933 |
| Has physically hurt you? | 11 (10.1) | 61 (8.4) | 0.527 |
| Forces you to participate in sexual   activities that make you feel   uncomfortable? | 10 (9.3) | 66 (9.0) | 0.941 |
| In the past 3 months have you been   physically assaulted? § | 14 (12.3) | 52 (7.1) | 0.035 |
| In the past 3 months, have you   been raped or forced to have sex? § | 5 (4.6) | 26 (3.6) | 0.583 |
| **Stigma** |  |  |  |
| How often have you heard that homosexuals are not normal?  Never  Once or twice  A few times  Many times | 29 (26.9)  10 (9.3)  26 (24.1)  43 (39.8) | 203 (28.0)  72 (9.9)  131 (18.0)  320 (44.1) | 0.514 |
| How often have you felt that you hurt or embarrassed your family because you have sex with men?  Never  Once or twice  A few times  Many times | 56 (52.3)  9 (8.4)  24 (22.4)  18 (16.8) | 412 (57.1)  74 (10.3)  105 (14.5)  131 (18.1) | 0.212 |
| How often have you been made fun of or called names because you have sex with men?  Never  Once or twice  A few times  Many times | 36 (33.3)  13 (12.0)  28 (25.9)  31 (28.7) | 367 (50.3)  88 (12.1)  133 (18.2)  142 (19.5) | 0.006 |
| How often have you been hit or beaten up because you have sex with men?  Never  Once or twice  A few times  Many times | 72 (66.7)  12 (11.1)  15 (13.9)  9 (8.3) | 625 (86.0)  49 (6.7)  33 (4.5)  20 (2.8) | <0.001 |
| How often have you had to pretend that you are not homosexual in order to be accepted?  Never  Once or twice  A few times  Many times | 37 (34.3)  11 (10.2)  32 (29.6)  28 (25.9) | 272 (37.5)  81 (11.2)  125 (17.4)  247 (34.1) | 0.019 |
| How often has your family not accepted you because you have sex with men?  Never  Once or twice  A few times  Many times | 64 (59.8)  10 (9.4)  15 (14.0)  18 (16.8) | 520 (72.1)  42 (5.8)  81 (11.2)  78 (10.8) | 0.061 |
| How often have you lost your friends because you have sex with men?  Never  Once or twice  A few times  Many times | 49 (45.4)  15 (13.9)  24 (22.2)  20 (18.5) | 402 (55.4)  74 (10.2)  119 (16.4)  131 (18.0) | 0.186 |
| How often have you been kicked out of school because you have sex with men?  Never  Once or twice  A few times  Many times | 90 (83.3)  5 (4.6)  7 (6.5)  6 (5.6) | 630 (86.7)  42 (5.8)  29 (4.0)  26 (3.6) | 0.448 |
| How often have you lost a place to live because you have sex with men?  Never  Once or twice  A few times  Many times | 80 (74.8)  11 (10.3)  8 (7.5)  8 (7.5) | 600 (82.8)  58 (8.0)  39 (5.4)  28 (3.9) | 0.183 |
| How often have you lost a job or career opportunity because you have sex with men?  Never  Once or twice  A few times  Many times | 83 (76.9)  8 (7.4)  9 (8.3)  8 (7.4) | 608 (83.8)  50 (6.9)  35 (4.8)  33 (4.5) | 0.227 |
| How often have you experienced police harassment because you have sex with men?  Never  Once or twice  A few times  Many times | 87 (80.6)  5 (4.6)  6 (5.6)  10 (9.3) | 628 (86.4)  37 (5.1)  38 (5.2)  24 (3.3) | 0.035 |

† Not all cells sum to N due to missing data.

‡ Chi-square test applied for categorical variables; Kendall’s tau applied for ordered categorical variables (e.g., PHQ); Fisher’s exact employed where cell size n<5; Wilcoxon rank sum test applied for continuous data comparisons, as all had non-normal distribution.

§Not contextualized to “in a relationship”
